# Platelet-Rich Fibrin Scaffolds for Cartilage and Tendon Regenerative Medicine: From Bench to Bedside

**DOI:** 10.3390/ijms20071701

**Published:** 2019-04-05

**Authors:** Silvia Barbon, Elena Stocco, Veronica Macchi, Martina Contran, Francesca Grandi, Alessio Borean, Pier Paolo Parnigotto, Andrea Porzionato, Raffaele De Caro

**Affiliations:** 1 Department of Neuroscience, Section of Human Anatomy, University of Padova, Via A. Gabelli 65, 35121 Padova, Italy; silvia.barbon@yahoo.it (S.B.); elena.stocco@gmail.com (E.S.); veronica.macchi@unipd.it (V.M.); martina.contran@gmail.com (M.C.); andrea.porzionato@unipd.it (A.P.); raffaele.decaro@unipd.it (R.D.C.); 2 LifeLab Program, Consorzio per la Ricerca Sanitaria (CORIS), Veneto Region, Via N. Giustiniani 2, 35128 Padova, Italy; 3 Complex Operative Unit—Pediatric Surgery, Hospital of Bolzano, Via L. Böhler 5, 39100 Bolzano, Italy; francesca.grandi7825@gmail.com; 4 Department of Immunohematology and Transfusion Medicine, San Martino Hospital, 32100 Belluno, Italy; alessio.borean@ulss.belluno.it; 5 Foundation for Biology and Regenerative Medicine, Tissue Engineering and Signaling (T.E.S.) Onlus, 35131 Padua, Italy; pierpaolo.parnigotto@unipd.it

**Keywords:** platelet-rich fibrin, platelet growth factors, biomaterials, cartilage regeneration, tendon regeneration

## Abstract

Nowadays, research in Tissue Engineering and Regenerative Medicine is focusing on the identification of instructive scaffolds to address the requirements of both clinicians and patients to achieve prompt and adequate healing in case of injury. Among biomaterials, hemocomponents, and in particular Platelet-rich Fibrin matrices, have aroused widespread interest, acting as delivery platforms for growth factors, cytokines and immune/stem-like cells for immunomodulation; their autologous origin and ready availability are also noteworthy aspects, as safety- and cost-related factors and practical aspects make it possible to shorten surgical interventions. In fact, several authors have focused on the use of Platelet-rich Fibrin in cartilage and tendon tissue engineering, reporting an increasing number of in vitro, pre-clinical and clinical studies. This narrative review attempts to compare the relevant advances in the field, with particular reference being made to the regenerative role of platelet-derived growth factors, as well as the main pre-clinical and clinical research on Platelet-rich Fibrin in chondrogenesis and tenogenesis, thereby providing a basis for critical revision of the topic.

## 1. Introduction

The development of cost-effective biomaterial scaffolds to regulate inflammation and enhance wound healing processes is one of the most intriguing challenges in modern regenerative medicine and tissue engineering (TE). Materials of both biological and synthetic origin have been extensively investigated for regenerative purposes. In particular, naturally derived biomaterials offer the advantages of receptor-binding ligand presentation and susceptibility to cell-triggered proteolytic degradation and remodeling [1].

**Figure 1:**
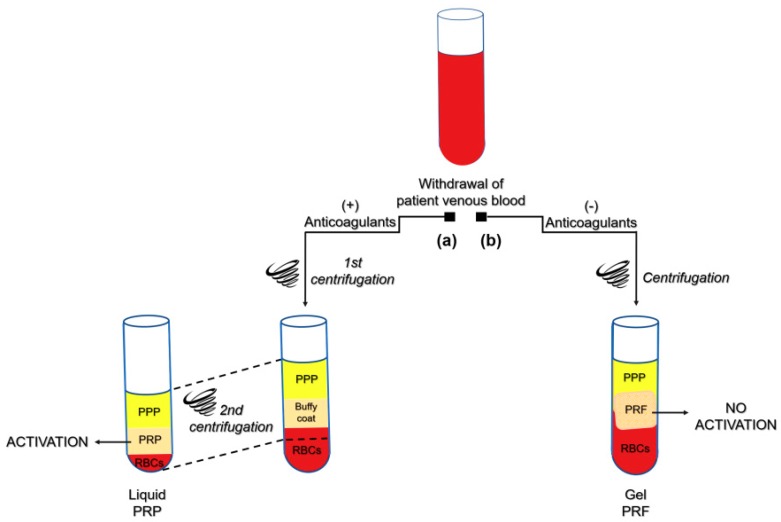
Platelet concentrates preparation protocols. Schematic drawing of the classical preparation protocols of PRP (Platelet Rich Plasma) and PRF (Platelet Rich Fibrin) hemocomponents. According to PRP protocol (**a**), blood is collected by venipuncture in the presence of anticoagulants. Thereafter, a two-step centrifugation procedure occurs. The first centrifugation yields three layers: RBCs (red blood cells), “buffy coat” and PPP (platelet poor plasma); hence, PPP and “buffy coat” are transferred into another tube and centrifuged again. After discarding the PPP fraction, the resulting PRP is suspended and activated by fibrin. As regards PRF protocol (**b**), venous blood is withdrawn without anticoagulants and centrifuged, causing the coagulation and stratification of blood components. In the middle of the tube, between the PPP and the RBC layers, a PRF clot develops, which naturally entraps platelets, leucocytes and molecules like growth factors and fibronectin. To be considered “platelet rich”, hemocomponents should be 5 times concentrated in platelets. Thus, the drawing is intended to be representative of the PRP and PRF manufacture steps, as many variations of the protocols are reported in the literature. There is general consensus in referring to Choukroun’s protocol [3] as the first method for PRF development.

Among natural biomaterials, Platelet-rich fibrin (PRF) has recently aroused widespread interest as a biophysical and biochemical milieu that delivers growth factors (GFs), cytokines and immune/stem-like cells for immunomodulation and tissue healing purposes [2]. PRF was first introduced by Choukroun and Collaborators [3] as a leukocyte- and platelet-rich biomaterial for oral and maxillofacial surgery applications. The preparation protocol consists of blood collection by venipuncture and subsequent centrifugation to form a strongly polymerized fibrin clot. The technique requires neither an anticoagulant nor thrombin/calcium gluconate [4] (Figure 1). Choukroun’s PRF has several advantages over Platelet-rich plasma (PRP), including the denser fibrin network, which allows for easier handling and suturing, as well as slower degradation rates after application, and consequently, delayed GFs and cells release profiles [5,6]. Moreover, this blood-derived membrane is enriched with leukocytes, which play a key role not only in immune and antibacterial responses, but also in the wound healing process [7,8]. Since Choukroun’s PRF was first described, many variations of the original protocol have appeared, resulting in the production of PRF-like materials with different architectures and cell contents [9,10,11,12,13,14,15,16,17]. The fundamental challenge to be overcome remains the concentration of platelets, which should be increased to a minimum of 5 times above baseline values for the hemocomponent to be considered “platelet rich” [18].

Regarding end-use destination, the PRF has been investigated recently in a wide range of medical fields including dentistry [19], oral implantology [20], maxillofacial surgery [21] and orthopedics [22]. Together with ease of availability and isolation, economical preparation method and good handling and storage properties, the possibility to prepare PRF of autologous origin minimizes the risks of immune rejection and pathogen transmission, paving the way for its safe use in regenerative medicine applications [23]. The therapeutic effect of PRF is mainly due to the high variety of platelet-derived protein molecules, which include signaling, membrane proteins, protein processing, cytoskeleton regulatory proteins, cytokines, and other bioactive peptides that activate and control the wound-healing signaling cascade [24]. It is well demonstrated that platelet proteome consists of 190 membrane-associated and 262 phosphorylated proteins, which were identified via independent proteomic and phosphoproteomic profiling [25]. Among bioactive molecules stored and released by platelet α-granules, the following GFs are the ones which are most commonly considered for tissue regeneration: platelet-derived GF (PDGF), insulin-like GF (IGF-1), transforming GF-β1 (TGF-β1), vascular endothelial GF (VEGF), basic fibroblastic GF (bFGF), and epidermal GF (EGF) [24].

Interestingly, beside the enrichment in platelets and leukocytes, the entrapment of stem-like cells with high regenerative potential within the fibrin network has recently been acknowledged [11,12], providing an even more solid basis for the use of PRF in regenerative medicine.

Recently, PRF regenerative application has been extended also to the field of cartilage and tendon repair. Platelet-derived GFs (i.e., PDGF, TGF-β1, IGF-1) can work as potent stimulators of chondrogenesis and tenogenesis by regulating cell proliferation, inflammation, neo-angiogenesis and extracellular matrix (ECM) deposition. The application of a single GF demonstrated a significant role in the enhancement of healing; however, because of dilution, the single “healing signal” exerts a temporary boost effect also on the outcome. This suggests the idea that administration of a pool of GFs gathered in an autologous product like the PRF may overcome such limitations [26]. 

Although many authors are now investigating the role of PRF in cartilage and tendon tissue engineering, the literature is still lacking a review of the most notable findings in the field. Thus, this work overviews the relevant advances in the use of PRF for cartilage and tendon regeneration, focusing on the role of GFs in tissue healing and pre-clinical and clinical application studies. 

## 2. PRF-GFs in Chondrogenesis

In cartilage regeneration studies, PRF is currently emerging as a biological tool to deliver supraphysiological levels of GFs and cytokines to the site of injury. Besides their physiological role, the in situ administration of platelet-derived GFs was demonstrated to stimulate in vitro proliferation and differentiation of chondrocytes [27] and to promote in vivo healing of cartilage [28]. Therefore, platelet GFs might offer promising treatments for the enhanced regeneration of focal articular cartilage defects.

The effect of platelet-derived GFs in chondrogenesis has been investigated both in vitro and in vivo by using platelet concentrates in the form of medium supplements or gels that encapsulate cells (i.e., chondrocytes, mesenchymal stem cells) [29,30]. Interestingly, Gaissmaier and Collaborators [31] demonstrated an increase of human chondrocyte proliferation rates following the addition of 1% and 10% human platelet supernatant to the culture medium. Similarly, the in vitro study performed by Akeda and Colleagues [32] highlighted the stimulating effect of 10% PRP-enriched medium on porcine chondrocyte proliferation, as well as collagen and proteoglycan biosynthesis. The positive effects of PRP on articular cartilage regeneration have been pointed out by several in vivo studies based on the administration of the hemocomponent in animal models of osteochondral defects, revealing an efficient capacity to promote cartilage healing [33,34]. Like PRP, PRF also contains a plethora of GFs and cytokines released from the platelets. Thus, it could positively influence articular cartilage regeneration through the same mechanisms described for PRP, ensuring superior mechanical performance which better meets the demands of the target tissue. 

Regarding the role of platelet-derived factors in chondrogenesis, PDGF is first GF present in a wound, where it serves to promote tissue healing by collagen and protein synthesis. It exhibits mitogenic activity to fibroblasts, vascular muscle cells, glial cells and chondrocytes [35]. In particular, PDGF has been reported to stimulate chondrocyte proliferation through the extracellular signal-regulated kinase 1/2 pathway [36], as well as inhibiting the IL-1β-mediated activation of NF-kB and cell apoptosis [37].

IGF-1 has been shown to regulate articular cartilage metabolism in both healthy and disease conditions. In vitro studies on normal chondrocyte cultures have demonstrated that this GF stimulates ECM synthesis and decreases matrix catabolism [28]. Moreover, IGF-1 is able to induce chondrogenic differentiation of Mesenchymal Stem Cells (MSCs), exerting even higher stimulation when used together with TGF-β1 [38]. In animal models of Osteoarthritis (OA), IGF-1 has demonstrated its potential to enhance cartilage repair and protect the synovial membranes from chronic inflammation [39]. However, the capacity of chondrocytes to respond to IGF-1 seems to decrease with age [40] and in OA [41].

TGF-β1 stimulates ECM synthesis by chondrocytes and inhibits the catabolic activity of IL-1 [42]. Both in vitro studies on MSC cultures and in vivo investigations on embryonic cartilage development have demonstrated the role of TGF-β1 in mediating the shift from proliferation to differentiation of chondrocytes [43]. Moreover, TGF-β1 has been shown to induce in vitro chondrogenesis of synovial lining and bone marrow-derived MSCs [44]. In vivo studies on rabbits provided evidence of TGF-β1-mediated repair of cartilage defects [45]. Recently, some authors have also described scaffold functionalization with TGF-β1 in order to deliver this bioactive factor to the site of cartilage injury [46,47].

bFGF can be found in relative abundance in the pericellular matrix of cartilage, and it is believed to play a key role in preserving the chondrocyte phenotype during expansion. On joint loading, its binding to cell surface receptors activates anabolic signaling pathways which lead to a decrease of aggrecanase activity, not affecting proteoglycan content [35]. In human OA, bFGF catabolic effect on dedifferentiation depends on the upregulation of the matrix metalloproteinases MMP1 and MMP13, and the downregulation of aggrecan and collagen II [48].

EGF signaling strongly influences chondrogenesis by upregulating the expression of SOX9 [49]. Due to this, EGF has been recognized as a potent mitogen of cultured chondrocytes [50]. In addition, an in vitro study on human articular chondrocytes have demonstrated that EGF treatment protracted extracellular signal-regulated kinases (ERK1/2) and Akt phosphorylation to regulate the chondrocyte ECM deposition [51].

Generally known as a potent stimulator of vasculogenesis and angiogenesis, VEGF has been found to be secreted by hypertrophic chondrocytes. Its angiogenic activity can attenuate hypoxic states, which is necessary for chondrocyte phenotype preservation [35]. The role of VEGF in chondrogenesis has been mostly investigated by knockout studies, demonstrating that it inhibits migration and accelerates proliferation of chondroprogenitor cells in vitro, as well as playing a crucial role in chondrocyte metabolism [52].

Clearly, the synergistic action of different GFs is needed to enhance and regulate chondrogenesis; based on that, the therapeutic use of PRF as a reservoir of several bioactive agents could constitute a valid strategy to accelerate the cartilage healing process.

## 3. PRF in Cartilage Tissue Engineering

Cartilage tissue engineering is mainly focused on the treatment of articular joint defects. Being an avascular and hypocellular tissue, articular cartilage (AC) shows limited intrinsic ability for self-repair, which implies that cartilage injuries cause progressive and degenerative joint diseases, such as OA [53].

Over the last two decades, several therapeutic strategies have been tested for AC regeneration, including scaffold-free approaches, cell-free methods, and advanced therapies associating cells with different biomaterials [54]. The first strategy relies on the only use of cells at high densities to promote cell-to-cell interactions and cell-based extracellular matrix neo-deposition. Autologous chondrocyte implantation represents the clinical option of choice [55,56], but it has recently been shown to be ineffective as a long-term treatment due to the dedifferentiation of chondrocytes during in vitro expansion [57]. Among alternative cell types that are under investigation for cartilage repair, MSCs from different sources (i.e., bone marrow, adipose tissue, umbilical cord) have gained considerable interest by virtue of their ease of isolation, high expansion capacity and chondrogenic differentiation potential [54,56,57]. Regarding cell-free strategies, the implantation of unseeded scaffolds made of both natural (collagen, hyaluronan, acellular matrices) and synthetic [poly(lactic-co-glycolic acid)] materials is combined with microfracture procedures [58] for the recruitment of bone marrow-derived MSCs to repair cartilage lesions [54]. Finally, the production of advanced tissue substitutes by associating cells and scaffolds seems to promise breakthroughs in the clinical practice of osteochondral regeneration [54]. In this regard, innovative strategies such as microfluidic biofabrication and cartilage bioprinting are offering unique possibilities to combine cells and biomaterials in an ordered and predetermined way for the manufacture of organized three-dimensional tissue constructs [54,59,60].

Despite progress in the field, the biological and functional outcome of most current treatments still remains controversial, mainly because the repaired tissue often shows fibrocartilaginous characteristics, not resembling the mechanical properties or ECM zonal organization of the native articular cartilage [61]. Thus, an intriguing challenge for orthopedic surgeons is represented by the development of an ideal treatment option which promotes hyaline cartilage neo-formation and subchondral bone preservation. From this perspective, the use of blood-derived products such as PRF has recently been recognized as a promising strategy to improve functional cartilage regeneration, by virtue of the presence of bioactive factors which stimulate the proliferation of chondrogenic cells and deposition of cartilaginous ECM [62].

### 3.1. In Vitro Studies

In vitro studies of the chondrogenic potential of PRF are quite scant (Table 1). This can probably be ascribed to the fact that the beneficial effects of platelet-derived GFs on chondrocyte and MSC proliferation, as well as on cell deposition of cartilaginous matrix, have been extensively demonstrated by in vitro investigation on PRP [63,64]. In the attempt to define the effect of PRF-derived GFs on chondrocyte proliferation and cartilage-specific ECM synthesis, Chien and Colleagues [65] investigated the incorporation of human PRF exudates into biodegradable fibrin (FB) scaffolds made from bovine fibrinogen and thrombin. In parallel, FB scaffolds alone and agarose (AG) scaffolds were used as controls. Before cell culture experiments, the concentrations of PDGF-BB, TGF-β1, IGF-1 and bone morphogenetic protein (BMP-2) into PRF exudates were quantified in comparison with FB scaffolds and three blood derivatives (i.e., serum, plasma and fibrin). Enzyme-linked immunosorbent assay (ELISA) demonstrated that the amounts of these GFs and cytokines were higher in PRF exudates rather than in the blood-derived products, except for TGF- β1. In this case, PRF exudates resulted to be richer in TGF-β1 when compared to serum and fibrin, but did not reach the concentration level of plasma samples. Subsequently, FB scaffolds +/− PRF exudates and AG scaffolds were seeded with primary chondrocytes from the knee cartilage of osteoarthritic patients and a human chondrosarcoma cell line, SW-1353. Both 2D- and 3D cultures of each cell type were tested. Overall, experimental data highlighted that cell growth rate, type-II collagen and Glycosaminoglycan (GAG) mRNA expression, as well as GAG and proteoglycan protein accumulations, were significantly increased when chondrocytes were cultured on FB scaffolds added with PRF exudates [65].

More recently, in vitro investigations based on rabbit chondrocyte cultures were carried out to evaluate the effect of an injectable PRF (i-PRF) on osteochondral regeneration [61]. Different from standard PRF, which consists of a 3D fibrin matrix, the development of the low speed centrifugation concept made it possible to obtain a new formulation of PRF for injectable purposes (i-PRF), to be used in clinical applications where this administration strategy is preferable. Interestingly, i-PRF can be injected similarly to PRP, with the advantage of forming a fibrin clot shortly after administration. Thus, this new formulation of PRF was compared to PRP by treating rabbit chondrocytes with culture media conditioned with 20% of each platelet concentrate releasates, in normal conditions or in the presence of interleukin (IL)-1β to mimic an osteoarthritic microenvironment. Gene expression studies on chondrocytes in normal conditions have demonstrated that i-PRF up-regulated chondrogenesis-related genes (SOX9, COL2A1 and ACAN) better than PRP. In the osteoarthritis-like environment induced by IL-1β, the inhibition of pro-regenerative genes (i.e., SOX9, COL2A1 and ACAN) was observed, coinciding with the up-regulation of genes related to arthritic disease progression (i.e., ADAMTS4, PTGS2 and MMP13). Remarkably, i-PRF addition to IL-1β-conditioned cultures has been shown to be superior to PRP treatment in modulating the inflammatory environment, by up-regulating the expression of pro-regenerative genes and down-regulating osteoarthritis-related markers [61].

A detailed characterization of PRF-induced chondrocytes harvested from rabbit cartilage was performed by Wong and Co-workers [66]. First of all, the PRF prepared from rabbit blood samples was characterized for (a) PDGF, IGF-1 and TGF-β1 release, (b) mechanical behavior, and (c) ultrastructural morphology by Scanning Electron Microscopy. After that, the chemotactic stimulus induced by PRF on chondrocytes was evaluated using both isolated cell populations and cartilage fragments co-cultured with the hemocomponent. In particular, cell exclusion zone and transwell migration assays revealed the ability of PRF scaffolds to attract and nourish chondrocytes during both in vitro and ex vivo tests. Moreover, chondrocytes were treated with PRF-conditioned media in different concentrations (25%, 50%, 100%), confirming that the hemocomponent increased cell proliferation rate in a dose-dependent manner. Finally, PRF was proven to up-regulate cell chondrogenic markers, such as type-I and type-II collagen and Aggrecan, as well as to stimulate the deposition of cartilaginous matrix (i.e., GAG accumulation) [66].

The chondrogenic potential of PRF-derived factors were assessed not only on chondrocyte cultures, but also on different cell types, such as rabbit meniscocytes [67] and human adipose-derived stem cells (ASCs) [68]. The work by Wong and Colleagues [67] on meniscocytes is very similar to the study on chondrocytes previously reported by the same research group [66]. Also, in this case, rabbit PRF demonstrated the ability to stimulate cultured meniscocytes in terms of cell migration, proliferation and deposition of cartilaginous matrix.

To the best of our knowledge, the only in vitro study on stem populations was reported by Souza and Colleagues [68] and considered the stimulation of human ASCs with eluates recovered from human Fibrin rich plasma (FRP) membranes. This treatment was successful in increasing cell proliferation in 2D cultures, as well as specific differentiation towards the chondrogenic lineage of micromass 3D cultures, as demonstrated by the induction of mucopolysaccharides and aggrecan synthesis.

### 3.2. Pre-Clinical Implantation

Often by passing the in vitro study phase, the pre-clinical implanting of PRF for cartilage repair has been evaluated by several Authors (Table 2). The hemocomponent was tested for the treatment of chondral [62,66,69,70,71,72], osteochondral [53,61,73,74,75,76] or meniscal [67] defects of the knee joint, as well as full-thickness cartilage defects of the ear [77]; the investigated animal models include rabbits [61,62,66,67,69,73,77], pigs [75], dogs [53,70,71,74,76] and horses [72].

Although PRF alone has been shown to positively influence cartilage repair in terms of the improvement of macroscopic and histological grading scores [70,71], most studies considered the possibility of implementing its regenerative potential by combining the implantation of the hemocomponent with (a) cartilage granules/fragments [66,69,75], (b) bone marrow-derived MSCs [53,72,74,76,77] and (c) the chemokine Stromal cell-derived factor (SDF)-1 [62]. In addition, PRF implantation was also investigated in association with other therapeutic strategies, such as suture repair of meniscal defects [67] and osteochondral autograft [73].

Interestingly, the liquid formulation of PRF, called i-PRF, was tested not only in vitro to improve chondrocyte proliferation and differentiation (see Section 3.1), but also in vivo, through the injection into rabbit models of osteochondral lesions [61]. Finally, rather than using PRF as a 3D matrix delivering regenerative cells and GFs, Wu and Colleagues [53] tested the addition of PRF releasates (PRFr) to MSC implantation for osteochondral defect repair.

Remarkably, some pre-clinical investigations also reported comparisons between PRF and PRP therapeutic effects on cartilage regeneration, mainly with the aim of verifying whether the fibrin network offered better structural support to cartilage and subchondral bone repair [61,62,71,73].

After PRF administration into the defect site, macroscopic and histological evaluations of repair tissue were the key parameters in the study of cartilage healing. In particular, the International Cartilage Repair Society (ICRS) scoring system was the main reference for both macroscopic and microscopic assessment of the regenerated articular cartilage [61,62,66,69,70,71,72,73,74,75,76]. Furthermore, some authors performed gene and protein expression studies of specific cartilage markers (i.e., type-I and type-II collagen, Aggrecan, Sox9) [62,73,77], as well as the evaluation of implant immunogenicity by determining blood levels of CD4/CD8, Interleukin (IL)-2 and IL-4 [77]. 

Overall, pre-clinical investigations revealed a significant improvement of cartilage regeneration after PRF/i-PRF/PRFr treatment, with general evidence of enhanced healing after combining the hemocomponent with autologous cartilage [66,69,75] or MSC administration [53,72,74,76,77]. In particular, ICRS macroscopic evaluation made it possible to attribute significantly higher scores to PRF-treated defects regarding degree of damage repair, integration with the border zone, regularity of repair tissue surface and overall repair grading [61,62,70,71,72,73,74]. In parallel, higher scores were registered also by a histological grading system, PRF-treated groups presenting normal cell distribution and cartilage mineralization, a higher number of chondrocyte-like cells, hyaline cartilage-like formation and no subchondral abnormalities, as well as good integration with the surrounding cartilaginous tissue [53,61,62,66,67,69,70,71,72,73,74,75,76]. According to these criteria, PRF proved to be superior to PRP in promoting cartilage healing in most cases [61,62,73]. Regarding chondrogenic marker expression, Aggrecan, SOX9 and type-II collagen resulted in up-regulation in the repair of cartilaginous tissue after PRF treatment [62,77]. Remarkably, staining for type-I and type-II collagen highlighted the fact that PRF better promoted the formation of hyaline-like cartilage rather than fibrocartilage in the defect site [73]. 

In addition to the orthotopic implant, the viability of cartilage grafts embedded in PRF has been evaluated through subcutaneous implant, from the perspective of applications in rhinoplastic surgery [78,79] (Table 2). Autologous cartilage grafting is a standard procedure in rhinoplasty, diced cartilage representing the most popular option [79]. In the effort to maintain its structure, function, and viability upon implantation, as well as to avoid tissue scattering and subcutaneous irregularities formation, diced cartilage graft is commonly wrapped into acellular cadaveric dermis (e.g., AlloDerm), oxidized regenerated cellulose (e.g., Surgicel) or autologous fascia. Although they are widely used in clinical practice, these materials still present some important drawbacks, such as high resorption rates, high costs and the need for a second incision [78,79]. In this context, PRF has been investigated as a bioactive material which could extend cartilage graft viability during rhinoplastic procedures of nasal dorsum augmentation/correction. To this end, diced rabbit cartilage was wrapped with autologous PRF and implanted into dorsal subcutaneous pockets of rabbit models. In parallel, diced cartilage wrapped with acellular cadaveric dermis, oxidized regenerated cellulose or autologous fascia were implanted for a direct comparison. The in vivo studies highlighted that PRF can ensure better cell viability of the cartilage graft with greater biocompatibility and less inflammation and fibrosis. Thus, PRF may represent an ideal autologous biomaterial for the transfer of diced cartilage grafts during rhinoplastic surgery [78,79].

Despite the satisfying outcome of in vivo animal studies, an important limitation in the current pre-clinical research is represented by the fact that PRF regenerative effect was mainly validated in rabbit models of osteochondral defects. As known, rabbits show high potential for spontaneous cartilage healing that is not seen in larger animal models and humans, losing favor with researchers for in vivo investigations of osteochondral repair [80,81]. Thus, pre-clinical studies on larger and more relevant animals (i.e., sheeps, pigs, dogs, horses) should be deepened to best highlight PRF efficacy and safety in experimental conditions which are as similar as possible to intended human use.

### 3.3. Clinical Trials

Although pre-clinical studies in pathological animal models is important to test the safety and biocompatibility of a scaffold, the final proof of regenerative potential is given by implanting in far more complex human patients. Regarding PRF, its clinical use could be prompted by the fact that it is mainly an autologous product which needs no/minimal manipulation before being implanted into the patient.

**Figure 2:**
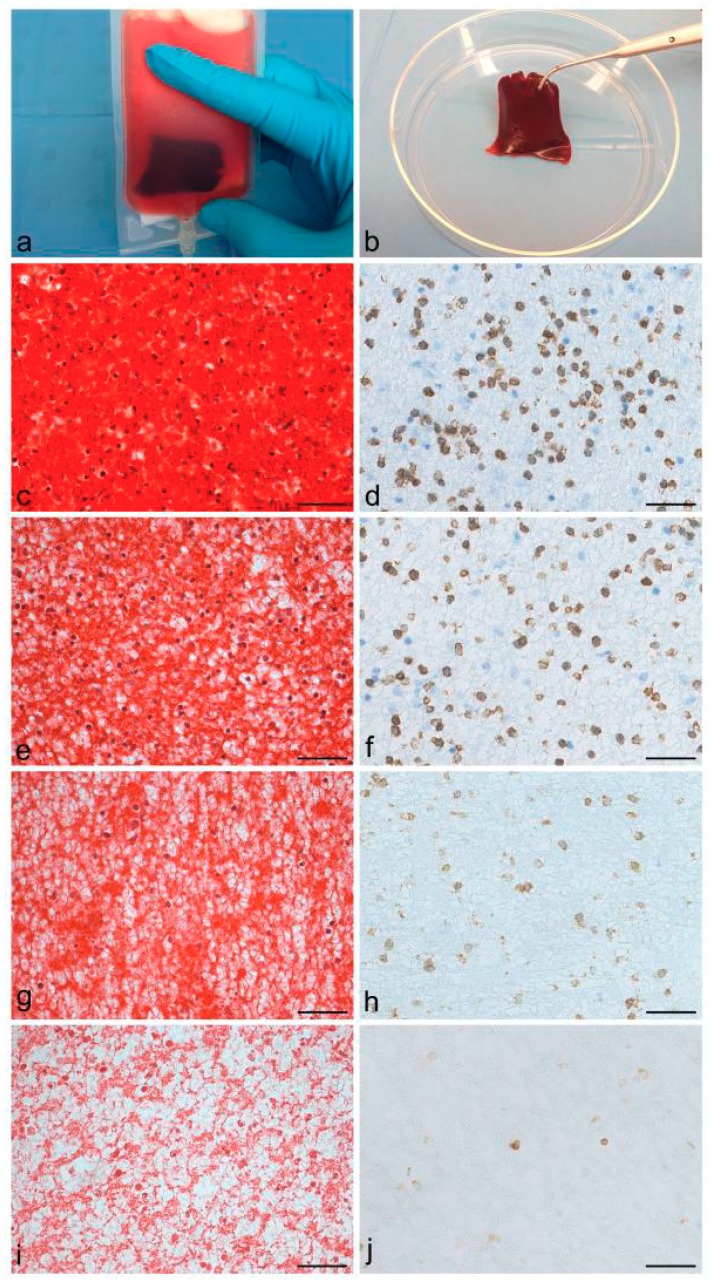
Macroscopic aspect of the leucocyte- and platelet-rich fibrin membrane (CLP-MB) soon after manufacture (**a**,**b**). Biebrich scarlet buffer-fuchsine acid staining (**c**,**e**,**g**,**i**) and immunological localization of CD3-positive cellular elements (**d**,**f**,**h**,**j**) on the CLP-MB after 4 (**c**–**d**), 7 (**e**–**f**), 14 (**g**–**h**) and 21 (**i**–**j**) days of PBS incubation at 37 °C. It is possible to recognize a progressive reabsorption of the fibrin matrix and loss of the cellular elements (**c**,**e**,**g**,**i**), as well as the reduction of lymphocytes (**d**,**f**,**h**,**j**). Scale bar: 37.5 µm.

To our knowledge, only four studies can be found in the literature about the clinical application of PRF for cartilage repair (Table 3). In 2015, Buda and Collaborators [82] reported a retrospective study about the treatment of hemophilic ankle arthropathy by the use of a collagen matrix seeded with bone marrow-derived cells (BMDCs) and added to autologous PRF. In hemophilic patients suffering from joint cartilage degeneration due to hemarthrosis, regenerative approaches, such as bone marrow-derived cell transplantation (BMDCT) and Matrix Autologous Chondrocyte Implantation (MACI) have poorly been considered [82,83]. The aforementioned study described the arthroscopic implantation in the ankle joint of a collagen membrane loaded with BMDCs and PRF. After a mean follow-up of 2 years, patients were evaluated by means of American Orthopaedic Foot and Ankle Society (AOFAS) scores, radiographs, magnetic resonance imaging (MRI) and Mocart scores, detecting signs of osteochondral regeneration and no progression of joint degeneration. Three of the five treated patients were also able to return back to sporting activities, leading to the conclusion that BMDCT coupled to PRF could represent a valid strategy for promoting cartilage restoration in mild ankle hemophilic arthropathy [82]. Two subsequent clinical trials aimed at testing PRF capacity to regenerate knee chondral defects. In both cases, a novel approach to cartilage repair was investigated by performing microfractures in combination with platelet concentrate administration. In particular, Papalia and Colleagues [84] described a retrospective clinical study on 48 patients treated by three methods: (i) microfractures and intra-operative administration of PRF; (ii) microfractures and post-operative injections of PRP and (iii) microfractures alone. After 2 and 5 year-follow ups, clinical scores [International Knee Documentation Committee (IKDC) functional scores, Visual Analogue Scale (VAS) pain], as well as MRI and Mocart scores revealed that the addition of platelet concentrates significantly improved microfractures outcome, with PRF ensuring better and earlier results than PRP [84]. Similarly, the work by D’Antimo and Collaborators [85] retrospectively investigated the regenerative effect of microfracture technique and the concomitant application of an autologous leucocyte- and platelet-rich fibrin membrane (CLP-MB). This hemocomponent is produced through blood collection by multiple apheresis cycles to obtain a leucocyte–platelet concentrate which is added to a plasma cryoprecipitate [9,10]. The final membrane is highly enriched with platelets, leukocytes, monocytes/macrophages, fibrinogen, and CD34^+^ cells. Our research group performed a detailed in vitro characterization of the CLP-MB membrane, highlighting its regenerative properties in terms of GF release, mechanical behavior and biodegradation profile [12]. The CLP-MB exhibits good elasticity and high deformation capacity, demonstrating itself to be suitable for implantation in the Knee joint for articular cartilage repair. Moreover, the membrane has been shown to sustain over time the release of GFs and cytokines (i.e., PDGF-BB, VEGF and IL-10) responsible for cell proliferation/differentiation, angiogenesis stimulation, as well as regulation of inflammatory condition. In vitro biodegradation studies showed good preservation of the fibrin network up to 21 days, with a progressive loss in cellular elements (Figure 2).

**Figure 3:**
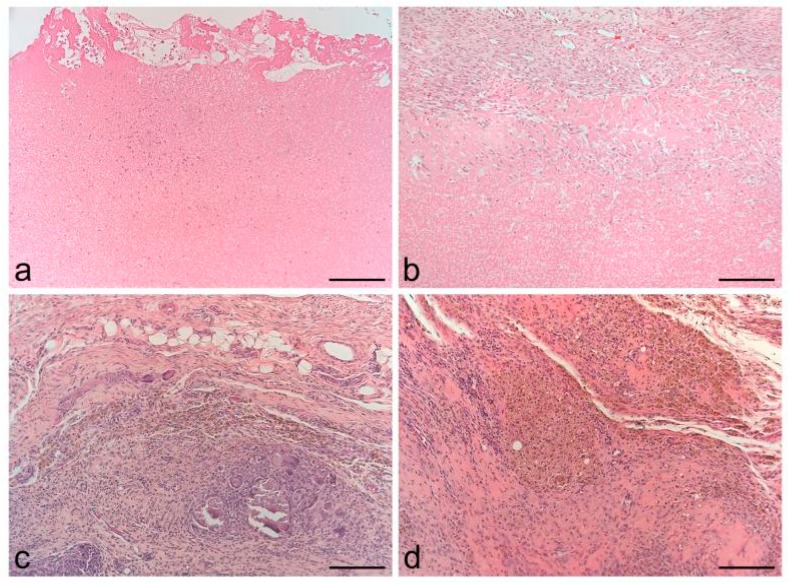
Hematoxylin and Eosin staining of the CLP-MB after 4 (**a**), 7 (**b**), 14 (**c**) and 21 (**d**) days of subcutaneous implantation into the dorsal region of athymic rats. A progressive reabsorption of the fibrin matrix is shown; in (**c**,**d**) the membranes are not easily detectable due to the almost complete biodegradation. Scale bars: 37.5 µm.

The subcutaneous implantation of CLP-MB into athymic rats demonstrated that it progressively biodegraded and was replaced by connective tissue, suggesting that it could work as a biomimetic scaffold which degrades with time to be substituted by neo-regenerated tissue (Figure 3) [12]. 

Regarding the clinical trial, 25 patients with focal chondral lesions underwent a single-step Autologous Matrix-Induced Chondrogenesis (AMIC) treatment. Briefly, after performing Steadman microfracture procedure [58], the CLP-MB membrane was placed upon the defect site and then covered by a homogenous layer of collagen-based injectable gel (Cartifill) [85]. During arthroscopic surgery, the CLP-MB membrane appeared to be easy to handle, adapting well to the cartilage lesion. After 1-, 6- and 12-month follow-ups, IKDC and VAS scores were considered to evaluate the short-term safety and efficacy of the therapy. Overall, clinical data showed that one-stage AMIC based on the implantation of CLP-MB with Cartifill significantly reduces pain perception and provides functional improvement. Of utmost importance, the preparation of CLP-MB for implantation was subject to quality control tests during all the production steps, assuring that a standardized, traceable and safe product was obtained [85].

PRF efficacy was clinically assessed not only in arthroscopic knee surgery, but also in rhinoplastic procedures for dorsal nasal augmentation purposes. Notably, Kovacevic and co-workers [86] experimented with, for the first time, a novel technique for cartilage graft stabilization by the use of autologous platelet concentrates. This retrospective clinical study presents the data collected from 48 patients subjected to rhinoplasty and implanted with heterogeneous grafts made of (a) cartilage scales, (b) a cartilage pâté, and (c) an autologous platelet concentrate to stabilize the construct. Interestingly, different platelet-rich products were used: liquid Plasma-Rich Growth Factors (PRGF) and PRF prepared according to two distinct protocols to obtain a liquid injectable PRF (i-PRF), or a so called advanced-PRF (a-PRF), prepared as a tear-resistant and flexible membrane. For PRGF, a few drops of the hemocomponent were released on the top of the cartilage scales-cartilage pâté compound graft, which was then incubated at 37 °C to improve fibrin polymerization. Regarding i-PRF, the platelet concentrate was sprayed on the top of the compound graft and completed coagulation in a few minutes, obtaining cartilage scales embedded in the fibrin matrix. The application of a-PRF was chosen when the graft was based only on cartilage pâté, which was placed on a layer of a-PRF and stabilized with i-PRF. This kind of graft was especially used for corrections of minimal dorsal irregularities or during tip surgery. Clinical data demonstrated satisfactory dorsal nasal augmentation in 47 cases, with only one patient showing graft displacement 3 months after surgery; however, no further displacement was registered. The implantation of a cartilage graft combined with platelet concentrates did not lead to dorsal irregularities, nor signs of resorption, erythema or inflammation, demonstrating itself to be a promising alternative to current surgical strategies for dorsal nasal augmentation [86].

## 4. PRF-GFs in Tenogenesis

To date, several strategies have been developed to ameliorate clinical outcomes in cases of acute or chronic injuries of the tendons; however, as yet, none is used routinely to treat patients, proving that tendon injuries are a significant clinical problem and that they remain a challenge in clinical practice [87]. In fact, tendons have the ability to heal naturally after injury, but with significant fibrosis that compromises mechanical outline and alters their in vivo performance, also making them more susceptible to further damage [88].

Even if tendons consist primarily of water and type-I collagen, with smaller amounts of other collagens and matrix materials and various types of cells, mainly fibroblasts [26], effective treatments are lacking, possibly due to a low level of comprehension of their biology with respect to that of other components of the musculoskeletal system. Hence, to understand the molecular basis of tendon regeneration, considering growth factors involved in tendon wound healing and adhesion may serve as a guide for customizing effective treatments based on the latest Regenerative Medicine methods [89].

As for other tissues, tendon injury is characterized by the onset of inflammation, cell proliferation, reparation by collagen deposition and ECM production up to remodeling [26]; these events are stimulated by the release of GFs and cytokines including interleukins, tumor necrosis factor (TNF), connective tissue growth factor (CTGF), VEGF, PDGF, bFGF, TGF-β1, EGF and IGF-1 [26,87,90]. 

Different roles have been recognized for GFs; they affect their own activity as well as the expression of other molecules. Considering their function, GFs support/control different stages of healing. IGF-1, PDGF and bFGF are essential during the early and intermediate stages of tissue regeneration as they aid fibroblasts in migration, proliferation and in synthetizing ECM. Regarding TGF-β1 and VEGF, they have some role in these processes too, but they are mainly involved in angiogenesis of the injured area where they regulate tissue remodeling [26]. However, embryological experiments and genetic analyses proved that TGF-β1 and bFGF are the main GFs with a role in tendon development [91,92]

GFs powerfully regulate cell biological responses; hence, their exogenous addition can further stimulate tissue recovery and the differentiation of stem cells into the tenogenic lineage [93]. Ectopic administration of a single GF has been considered for tendon healing but administering a pool of bioactive molecules may boost regeneration. According to this assumption, hemocomponents have been investigated.

The eminent role of IGF-1 in tenogenesis has been supported by much evidence. It increases collagen expression in adult human tenocytes in vitro and also collagen content and fibril diameter in tendon constructs [94]. However, its temporal expression in cases of injury has been debated. In a study by Dahlgren et al. [95], IGF-1 levels decreased of about 40% in the first 2 weeks in a horse model of lesioned flexor tendon compared to normal tendon; and they increased to exceed those of normal tendon at week 4. Conversely, other authors claimed to have identified IGF-1 during all phases of healing, even if it was mostly present in the early period of tendon development, highlighting its importance in the preliminary stages of regeneration. Yet, the idea that the exogenous administration of IGF-1 after injury may exert therapeutic advantages, i.e., by enhancing the metabolic response of tendon fibroblasts, is commonly accepted. It appears to aid the migration and proliferation of fibroblasts, also stimulating gene expression of collagen, and protein and extracellular matrix synthesis both in vivo [94] and in vitro [96]. This evidence confirms previous data by Murphy and Nixon [97], also observing in an in vitro study on equine tendon that IGF-1 acts in a dose-dependent manner.

At the time of tendon injury, PDGF peaks rapidly, being released from degranulating platelets; Therefore, it is thought to play a significant role in the early stages of healing [98]. It has many functions, including fibroblast mitogenesis, angiogenesis and activation of macrophages. Interestingly, it also promotes the production of other GFs including TGF-β1, IGF-1 and VEGF, confirming its leading role in healing processes [96]. 

According to many in vitro and in vivo studies, bFGF is involved in regulating cell proliferation and migration in addition to stimulating angiogenesis and collagen synthesis [26,96]. It is produced by normal tendons fibroblasts; moreover, its expression increases at the injured sites in various animal models of tendons injury [91].

TGF-β1 is produced by most cells involved in the healing process [26], and it is similarly active throughout the injury recovery period; in particular, TGF-β1 increases fibroblast stimulation, cell proliferation and collagen production, but it also recruits macrophages [96].

VEGF is only relevant toward the end of the inflammatory process [96], as confirmed by Petersen et al. [99], showing that its levels are neglectable in normal human Achilles tendons and increase in cases of rupturing. It is released by a variety of cells including platelets and tenocytes, thereby exerting a prominent role in wound healing as it initiates angiogenesis [90]. Conversely, with experimental tendon healing, it seems to have a rather deleterious effect, perhaps because of its angiogenic activity and by stimulation of matrix metalloproteinases [100].

## 5. PRF in Tendon Tissue Engineering

In perusing the literature, the potential effectiveness of PRF in vitro [101,102,103] or the outcomes of its pre-clinical or clinical use, have been investigated. Rotator cuff tears [104,105,106,107,108,109,110,111,112], ruptures of the Achilles tendon [101,113,114], flexor tendon healing [115,116], patellar tendon defects and medial collateral ligament reconstruction [117,118], as well as repair of gluteus medius tendons [119], are the preferred end-use destinations considered by the authors. 

### 5.1. In Vitro Studies

Surprisingly, in vitro studies describing the use of PRF are rare in comparison to pre-clinical or clinical trials (Table 4). However, solid in vitro evidence could be useful to understand the mechanisms underlying successful or unsuccessful therapies with PRF which, to date, have not found consensus among clinicians. 

To succeed in the reconstruction of injured ligament, controlled administration is required of chemotactic, mitogenic, and angiogenic factors that will promote cell metabolism, the formation of new blood vessels, and tissue remodeling [95]. To our knowledge, only Anitua and colleagues [101] have combined a preliminary in vitro to an in vivo study evaluating the effectiveness of autologous fibrin matrices. In particular, the authors compared the behavior of freshly isolated human tendon cells on homologous fibrin, platelet-rich (PR-) and platelet-poor (PP-) matrices. The platelets entrapped in the fibrin matrices boosted tendon cells proliferation, also increasing the synthesis of structural ECM-protein collagen-I, with no difference between PR- and PP-matrices. This result was unexpected, considering that TGF-β1 levels were higher in PR-matrix samples (i.e., with or without tenocytes) than PP-matrices. Probably, other platelet-released molecules, including hepatocyte growth factor (HGF) (i.e., a mitogen for endothelial cells [120]), may hide the effect of TGF-β1. Additionally, cultured tenocytes also synthetized VEGF and HGF; VEGF, but not HGF, was significantly higher in the presence of platelets. The eminent and debated role of TGF-β1, which stimulates VEGF secretion by tendon cells but also drives fibrogenesis up to scar tissue deposition, was later considered also by Visser et al. [102]. Three different hemocomponents, namely PRF matrix, PRF membrane and whole blood clot, were analyzed. Both PRF-derived constructs, characterized by dense fibrin scaffold, allowed for increased concentrations of eluted TGF-β1 and tendon cells proliferation. Evidence about the deep relationship between the physical form of the hemocomponent and GFs release was also highlighted by other Authors. In fact, Zumstein et al. [111] proved that matrices based on leukocyte- and platelet-rich fibrin (L-PRF) can guarantee higher levels of eluted GFs than normal blood and thus compared their release from a standard/gelatinous matrix versus a dry/compressed matrix. L-PRF clots showed a continuous slow release with an increase in the absolute release of growth factors TGF-β1, VEGF and myeloperoxidase-content (MPO) in the first 7 days in vitro; for IGF-1, PDGF-AB and platelet activity factor (CXCL4) it boosted in the first 8 h reaching zero at 28 days. Moreover, CXCL4, IGF-1, PDGF-AB, and VEGF were released in high levels from the standard/gelatinous matrix compared to the dry/compressed matrix; this represents behavior not encountered for MPO and the TGF-β1.

In addition to its role as a GFs delivery system, PRF may be also considered as a scaffold for cells adhesion and proliferation. In particular, considering the potential of MSCs in tissue regeneration, Beitzel et al. [103] evaluated their response to five different scaffolds: fresh-frozen human rotator cuff tendon (i.e., allograft); human highly cross-linked collagen membrane (Arthroflex; LifeNet Health, Virginia Beach, VA); porcine non-cross-linked collagen membrane (Mucograft; Geistlich Pharma, Lucerne, Switzerland); human platelet-rich fibrin matrix and fibrin matrix based on platelet-rich plasma (ViscoGel; Arthrex, Naples, FL). Even if MSC differentiation successfully occurred, adhesion was significantly greater to both the non-cross-linked porcine collagen scaffold and platelet-rich fibrin matrix. Considering proliferation, the non-cross-linked porcine collagen scaffold was much more effective compared with PRF-M and fibrin matrix based on platelet-rich plasma. Thus, stiffness of the matrix significantly affects cell behavior, suggesting the importance of preliminary studies dedicated to the development of the manufacture protocol. The role of the matrix is not only that of a mere surface; its intrinsic characteristics modulate cell behavior, and its mechanical properties have an additional, instructive role that, in the case of PRF, can also descend by its ability to entrap/release platelets and/or growth factors.

### 5.2. Pre-Clinical Studies

The literature reports on pre-clinical studies aiming to develop new approaches PRF-based towards, Achilles tendon rupture [101,114], regeneration of the tendon-bone insertion [108], healing of flexor tendon [115,116] and patellar tendon [117], also including medial collateral ligament reconstruction [118]. 

Different animal models of injuries were considered; the first experimental works were carried out on species with a complex neurological development, including sheep [101,114] and dogs [117]. Later, ethic issues, a growing awareness of the value of responsible animal research, together with mature surgical experience with the use of hemocomponents for regenerative purposes, have prompted researchers to favor species with simpler neurological developments, like rabbits [115,116,118] and rats [108].

The authors described the manufacture of different PRF-derived products according to the content in platelets and/or structure, but a shared preparation-protocol, as well as a strong characterization study to allow for comparisons, is lacking. Visser et al. [117], Hasan et al. [108], Liao et al. [116] studied PRF; Anitua et al. [101] considered both platelet-rich and platelet-poor matrices; others included plasma in the fibrin matrix (i.e., platelet-rich plasma fibrin matrix—PRPFM) [114,115] and then compared its healing effect versus PRP [115], while Matsunaga et al. [118] described a novel protocol to prepare a Compact Platelet-Rich Fibrin Scaffold (CPFS) resembling a sheet. Concerning surgical practice, hemocomponents were used either alone as a booster of tissue healing after surgery [101,108,115,116,117,118], or combined to an acellular porcine dermal (APD) patch [114]. 

Interestingly, regarding the origin of hemocomponents, both autologous [101,114,116,117] and allogenic preparations [108,115,118] were investigated. Assessing the effectiveness of allogenic products verifies the efficacy of these therapeutic approaches, even in patients for whom there are no clinical indications for autologous preparations (e.g., thrombocytopenic patients). Thus, an optimal characterization study may identify the quality parameters needed to assure the clinical efficacy of such products. As reported in Table 5, many different aspects were considered to evaluate the role of hemocomponents in tissue regeneration. In particular, newly-formed tissue was assessed for cell density and tissue morphology, vascularization, absence of inflammation, matrix deposition, adequate integration of the scaffold without adhesions, thickness of the repaired tissue, and the mechanical parameters. 

Both Anitua et al. [101] and Sato et al. [115] proved the effectiveness of fibrin matrices to enhance healing and the remodeling of tendons without side effects (i.e., edema or adhesions), to reduce risk of repeated rupture after surgery and to help in early rehabilitation. A positive outcome in using fibrin products was later suggested also by Matsunaga et al., [118] showing that CPFS accelerates healing of tendons and ligaments, acting as a provisional support for graft augmentation. Conversely, to gain regeneration in an injured Achilles tendon model, no decisive role of fibrin matrix was highlighted by Sarrafian et al. [114]. In fact, the authors, who mainly focused on a porcine dermal patch, observed that the addition of PRPFM to APD did not exert a substantial role in regeneration. A negative outcome after using fibrin products was also found. Visser et al. [117] claimed that PRF membrane did not enhance the rate or quality of tendon healing in cases of defects, also increasing the amount of repair tissue within and surrounding the defect. Similarly, the study results of Hasan et al. [108] showed that PRFM did not make it possible to recapitulate the native enthesis, but rather, induced a disorganized healing response characterized by fibrovascular scar tissue. Also, Liao et al., [116] reported undesirable effects on the biomechanical properties of repaired tendon, suggesting that sometimes PRF may hinder, rather than enhance, healing.

Besides the different pre-clinical study outcomes, which are likely due to different preparation protocols, the lack of data on functional recovery of the operated limb is also evident. The authors mainly focused on the quality of the regenerated tissue from both a histological and biomechanical perspective; however, assessments on mobility could provide interesting and useful data for a complete evaluation of the efficacy of fibrin-based products too.

### 5.3. Clinical Studies

The scientific rationale behind the use of PRF-based products is related to the intrinsic nature of the entrapped platelets, acting as a reservoir of many GFs; they accelerate the healing process, controlling pain and inflammation [21]. In addition, PRFs are easy to obtain, and so are promptly available for clinical use. Despite this, controversies in the literature regarding the benefits and the clinical outcomes cannot be neglected (Table 6).

Considering tendons, PRF has been mainly adopted to treat rotator cuff injuries [104,105,106,107,109,110,112]. Recently, in 2017, Alviti et al. [113] considered their use for acute ruptures of the Achilles tendon, while, Saltzman et al. [119] addressed the repair of the gluteus medius tendon. In all these clinical trials, PRF was used as an additional treatment to standard suture bridging (i.e., single or double row), and clinical outcomes were compared to those of routine-surgery approaches or, in a study by Alviti et al. [113], to the characteristics (i.e., gait analysis) of healthy subjects, suggesting the need for a strategy assuring a functional recovery which superimposable to that of normal tendon. In fact, demographic data about patients enrolled for surgery revealed the involvement of a working-age population, with an average age of about 53 and a lower range of 30. 

After surgery, follow-ups were variable, according to the specific parameter being considered. In particular, the outcome was evaluated taking advantage from outcome scales (i.e., ASES, American Shoulder and Elbow Surgeons; L’Insalata; Rowe; SANE, Single Assessment Numeric Evaluation; SST, Simple Shoulder Test; WORC, Western Ontario Rotator Cuff Index; UCLA, University of California, Los Angeles; ROM, Range Of Motion; FF, Forward Flexion; ER, external rotation; SANE, Single Assessment Numeric, Evaluation; SSV, Subjective Shoulder Value; Constant scale; VAS, Visual Analog Scale) [104,105,106,107,109,110,112]; MRI analysis [104,105,106,107,110]; Power Doppler Ultrasound [109]; gait analysis [113]; operative time [106,110] and narcotic consumption [110] or demographic variables [119].

As for both in vitro and pre-clinical studies, the results obtained from tendon surgery with PRF-based products were different. Most authors asserted that the use of PRF-based products does not play a key role in tissue healing, pain or re-tear rate, and also, that functional outcome scores were not improved [105,107,110,112,119]. However, as suggested by Castricini et al. [107], this may depend on tear size and the heterogeneity of available PRF preparation products. Only two studies expressed a negative opinion on PRF products according to regression analysis and re-tear rate data [106,109]. Conversely, Alviti et al. [113], through a gait analysis study, demonstrated that the approach by suture and PRF augmentation in acute ruptures of the Achilles tendon is responsible for improvements without differences with respect to the healthy-control group.

Operative time was considered only by two groups [106,110], and discrepancies were pointed out; however, this parameter is deeply related to both the surgeon’s ability and experience, as well as to the specific clinical-case, so it may only act as a general indicator. Finally, infections were a sporadic event; only Bergeson et al., [106] described their occurrence in 2 of 16 patients implanted with a platelet-rich fibrin matrix.

Considering the overall results, the major limitation of these studies is attributable to the low number of individuals included therein; thus, a factual and critical analysis of PRF effectiveness requires not only more careful attention to the manufacture protocol, but also the possibility to involve more candidates for surgery to assure the feasibility of an accurate statistical analysis.

## 6. Conclusions

To date, the literature demonstrates the interest of scientific community for the safe therapeutic approach represented by platelet-rich hemocomponents, which can be prepared as inexpensive autologous products with high regenerative potential. In particular, PRF is considered as a second-generation platelet concentrate which may offer many advantages over first-generation PRP, by virtue of its three-dimensional fibrin matrix, ensuring favorable mechanical properties and the prolonged release of GFs, cytokines and regenerative cells. This defines PRF as a true biomaterial delivering the key players of the healing process, with improved potential for application in a wider range of clinical fields. Nevertheless, one of the main issues to deal with in the clinical translation of PRF is the great variability of preparation protocols and equipment [121,122], which impedes the obtainment of a unique and standardized product. In addition, PRF biomaterials have an intrinsic variability depending on a multitude of factors. Autologous hemocomponents prepared with the same system but at different times may yield different results in bioactive factors content [122]. The peculiar characteristics of the patient/donor (i.e., life style, contingent or chronic disease including drugs treatments) affect the final product properties and responses to the treatment. For example, data about PRF from patients suffering from coagulation disorders treated with proper medicines (i.e., heparin, warfarin or platelet inhibitors) are scant [123]. Moreover, as recently assessed by Xiong et al. [124], age and sex may also be considered as variables [125,126]. With this in mind, better standardization of manufacturing procedures allowing for consistent outcomes to be predicted is urgently needed.

Regarding the therapeutic effects of PRF, the synergistic action of platelet GFs is recognized to be the key element for enhancing and regulating tissue regeneration. Thus, a detailed characterization of PRF releasates and their mechanism of action (i.e., cell signaling pathways) seems to be recommended to better understand their potential for clinical application. As an important gap in knowledge, the literature is lacking specific biomolecular characterization of PRF products, with a few studies trying to describe their content, but which are only focused on platelets counts and main GFs quantifications [12]. PRF appears to be very difficult to characterize, as it is a complex blood-derived preparation that concentrates hundreds of regenerative elements hard to be singularly investigated [127]. Also considering the heterogeneity of PRF products, developing a method to specifically identify their composition in terms of cell content, type and concentration of the released GFs/cytokines, as well as fibrin matrix fibers, would help to predict the therapeutic outcomes of hemocomponent clinical implantation.

Despite the aforementioned concerns which need to be addressed, the therapeutic efficacy of PRF has recently gained attention, also in the field of cartilage and tendon TE, which is still seeking ideal bioactive materials promoting adequate functional tissue recovery. Although the stages of healing are similar for various tissues, for cartilage and tendons, the regeneration process naturally occurs at a slower rate than that of other connective tissues, probably because of their dense and hypocellular composition. As it gathers all molecules that are physiologically involved in the healing response, autologous PRF is potentially useful to recover all tissue integrity after injury, and it would be of prime importance for tissues like cartilage and tendons, showing an intrinsic slow or often inadequate regeneration ability resulting in fibrosis.

The reviewed studies reporting PRF-mediated chondrogenesis or cartilage healing demonstrate a large consensus on the beneficial effects exerted by the hemocomponent on cartilaginous tissue regeneration. We found general agreement on the preparation protocol, referring to Choukroun and Collaborators [3] in most of the reported studies. The characterization of PRF composition and biomechanics, as well as in vitro assessment of its chondrogenic potential, appeared to be neglected; nevertheless, the therapeutic outcome of hemocomponent implantation for the treatment of full-thickness osteochondral defects has been proven to be generally successful by pre-clinical research and clinical trials. 

Conversely, studies regarding the use of PRF for tendon repair are often conflicting, showing different results in animal models and clinical trials. As previously stressed, a possible explanation for this variability could be the lack of a standardized preparation protocol.

However, apart from result variability, which could be district-dependent, the use of PRF in cartilage and tendon regenerative medicine is paving the way for new therapeutic strategies which may overcome the limits of the current surgical approaches. Although further in vitro and in vivo investigations are required to fully estimate the potential benefit of this new regenerative technology, the autologous origin and graft versatility of PRF, as well as its high content in regenerative elements, seem to be strongly encouraging and worthy of further, in-depth exploration.

As for future perspectives, a noteworthy application of PRF and its released GFs could be represented by the functionalization of biosynthetic scaffolds to improve their capacity to support cell adhesion and proliferation, as well as to promote functional cartilage and tendon regeneration. Also, the manufacture of composite scaffolds mixing or combining polymers and the PRF as different layers may be an intriguing and vanguard manner of working with this hemocomponent.

## Figures and Tables

**Table 1:** In vitro studies of PRF chondrogenic potential.

Hemocomponent/Experimental Groups	PRF Preparation Protocol	Characterization Parameters	Major Findings	Reference
- Human PRF exudates incorporated into Biodegradable Fibrin (FB) scaffolds *Controls:* - Bovine Biodegradable Fibrin scaffolds - Agarose scaffolds	Preparation according to Choukroun et al., 2001 [3]: - Blood collection without anticoagulant - Centrifugation (400 X g, 10 min) - Formation of a fibrin clot rich with platelets (PRF) in the middle of the tube, between the red blood cells and the acellular plasma	- Quantification of PDGF-BB, TGF-β1, IGF-1 and BMP-2 into PRF exudates - 2D and 3D cultures of human primary chondrocytes and a human chondrosarcoma cell line (SW-1353) - Proliferation studies - mRNA expression of type-II collagen and GAGs - Synthesis of GAGs and proteoglycans	When chondrocytes were cultured on FB scaffolds added with PRF exudates: - cell growth rate was significantly increased - mRNA expression of type-II collagen and GAGs was up-regulated - Synthesis of GAGs and proteoglycans was enhanced	Chien et al., 2012 [65]
- Rabbit i-PRF *Control:* - Rabbit PRP	- Blood collection without anticoagulant - Centrifugation (60 X g, 3 min) with Choukroun PRF Duo Centrifuge (Process for PRF, Nice, France) - Collection of the upper plasma layer designated as i-PRF	- i-PRF- and PRP-conditioned cultures of rabbit chondrocytes in normal conditions or in the presence of IL-1β - mRNA expression of chondrogenesis-related genes (SOX9, COL2A1 and ACAN) and osteoarthritis-related markers (ADAMTS4, PTGS2 and MMP13)	i-PRF was found to be superior to PRP in: - up-regulating chondrogenesis-related genes in normal conditions - counteracting IL-1β inflammatory effects in osteoarthritis-like environment	Abd El Raouf et al., 2017 [61]
- Rabbit PRF	Preparation according to Choukroun et al., 2001 [3]	- Quantification of PDGF, IGF-1 and TGF-β1 release - Mechanical tests - Ultrastructural morphology by SEM - In vitro and ex vivo evaluations of PRF chemotactic effect on rabbit chondrocytes - Proliferation of chondrocyte cultures - mRNA expression of cartilage markers (type-I and type-II collagen and Aggrecan) - GAG deposition	- PRF improved the chemotaxis, proliferation, and viability of the cultured chondrocytes - Chondrogenic markers were up-regulated in cell populations cultured with PRF-conditioned media - PRF increased the formation and deposition of the cartilaginous matrix produced by cultured chondrocytes	Wong et al., 2017 [66]
- Rabbit PRF	Preparation according to Choukroun et al., 2001 [3]	- PRF chemotactic effect on rabbit meniscocytes (scratch migration and transwell migration assays) - Cell proliferation - Histological evaluation of type-I and type-II collagen, Aggrecan and GAG deposition	- PRF stimulated cellular migration and proliferation of meniscocytes - Extracellular matrix synthesis by cultured meniscocytes was enhanced by treatment with PRF releseates	Wong et al., 2017 [67]
- Human FRP membrane	- Blood collection without anticoagulant but with a clot activator - Centrifugation (770× *g*, 12 min) - Pression of the fibrin clot with stainless steel plate (Box PRF BmdCon^®^) for exudate extraction - FRP membrane formation	- Proliferation of human ASCs - Differentiation of ASC micromass cultures towards the chondrogenic lineage	- FRP membrane eluates stimulated the proliferation of ASCs - Treatment with eluates induced mucopolysaccharide and aggrecan synthesis by differentiated ASCs	Souza et al., 2017 [68]

ASCs, Adipose-derived Stem Cells; BMP-2, Bone Morphogenetic Protein-2; FRP membrane, fibrin-rich plasma membrane; GAGs, Glycosaminoglycans; i-PRF, injectable PRF; IGF-1, Insulin-like Growth Factor; IL-1β, Interleukin-1β; PDGF-BB, Platelet-derived Growth Factor-BB; PRF, Platelet-rich Fibrin; PRP, Platelet-rich Plasma; SEM, Scanning Electron Microscopy; TGF-β1, Transforming Growth Factor- β1.

**Table 2:** Pre-clinical application of PRF for cartilage repair.

End Use Destination	Hemocomponent/Experimental Groups	PRF Preparation Protocol	Characterization Parameters	Major Findings	Reference
Rabbits Chondral defect in the femoral condyle (diameter: 3 mm; depth: 0.5 mm)	Rabbit PRF combined with cartilage granules derived from the created defect *Control:* - cartilage defect with no implantation	Preparation according to Choukroun et al., 2001 [3]: - Blood collection without anticoagulant - Centrifugation (400 X g, 10 min) - Formation of a fibrin clot rich with platelets (PRF) in the middle of the tube, between the red blood cells and the acellular plasma	- 3-month implantation - MRI - ICRS Visual Histological Assessment Scale (distribution of cells, mineralization of cartilage, tissue surface and matrix, cell population viability, subchondral bone abnormalities)	- Less cartilage degradation in the PRF-treated group according to the MRI T2 values - Better histological scores in the PRF group, presenting normal cell distribution and cartilage mineralization, smooth and continuous tissue surface, hyaline cartilage-like formation and no subchondral abnormalities	Kuo et al., 2011 [69]
Dogs Full thickness articular cartilage defect in the femoral condyle (diameter: 6 mm; depth: 5 mm)	Dog PRF *Control:* - cartilage defect with no implantation	Preparation according to Choukroun et al., 2001 [3]	- 4-, 16- and 24-week implantation - ICRS evaluation score for macroscopic assessment of the repaired tissue - O’Driscoll histological grading scale for microscopic investigation	- Formation of cartilage-like reparative tissue in both experimental groups, with higher number of chondrocyte-like cells and better ECM deposition in the PRF groups - Macroscopic and histological grading scores were found to be higher in the PRF-treated groups, indicating a better quality of cartilage repair	Kazemi et al., 2014 [70]
	- Dog L-PRF - Dog L-PRP *Control:* - cartilage defect with no implantation	Preparation according to Choukroun et al., 2001 [3]	- 4-, 16- and 24-week implantation - ICRS evaluation score for macroscopic assessment of the repaired tissue - O’Driscoll histological grading scale for microscopic investigation	- No significant difference in macroscopic scores between L-PRP and L-PRF treated defects, but lower scores in the untreated control group - High quality repair tissue in both L-PRF and L-PRP treated groups according to histological evaluations	Kazemi and Fakhrjou, 2015 [71]
Rabbits Subcutaneous implant to test graft viability for rhinoplasty	- Diced rabbit cartilage wrapped with rabbit PRFM - Diced rabbit cartilage wrapped with acellular dermal tissue - Diced rabbit cartilage wrapped with oxidized methylcellulose - Diced rabbit cartilage alone	Preparation according to Choukroun et al., 2001 [3]	- 10-week implantation - Histological stainings - Graft evaluated for chondrocyte viability, collagen content, ECM fibrillar structure and changes in peripheral tissues	- Better preservation of cartilage graft viability in the PRFM group - Less fibrosis, higher chondrocyte viability, better ECM deposition and less inflammation in the PRFM group	Güler et al., 2015 [78]
Rabbits Subcutaneous implant to test graft viability for rhinoplasty	- Diced rabbit cartilage wrapped with rabbit PRF - Diced rabbit cartilage wrapped with oxidized regeneratedcellulose - Diced rabbit cartilage wrapped with fascia - Diced rabbit cartilage alone	Preparation according to Choukroun et al., 2001 [3]	- 2 month-implant - Macroscopic evaluation - Histological staining - Explants evaluated for graft viability, fibrosis, inflammation and vascularization	- Superior viability of the cartilage graft wrapped with PRF in comparison with the cartilage graft wrapped with oxidized regenerated cellulose - No significant differences among the other groups - The 4 groups were not significantly different in terms of inflammation rate, fibrosis and vascularization	Göral et al., 2016 [79]
Rabbits Full thickness articular cartilage defect in the patellar groove (diameter: 4 mm; depth: 3 mm)	- Rabbit PRF - Rabbit PRP - Rabbit PRF + rhSDF_1_ - Rabbit PRP + rhSDF_1_ - Gelatin + rhSDF_1_ *Control:* - Untreated cartilage defect	Preparation according to Choukroun et al., 2001 [3]	- 4-week implantation - ICRS scores for macroscopic evaluations - ICRS Visual Histological Assessment Scale - Immunofluorescence analysis of type-II collagen expression - Gene expression study of cartilage markers (Aggrecan, SOX9)	- Higher ICRS macroscopic scores in the PRF + rhSDF_1_ group, with complete repair and good integration with the surrounding cartilage - ICRS histological scores of treated groups, except for the PRP group, were significantly higher than the untreated control - Neo-cartilages highly positive to type-II collagen in the PRF + rhSDF_1_, PRP + rhSDF_1_ and Gelatin + rhSDF_1_ groups - Higher expression of SOX9 in the regenerated tissue of all treated groups than the control group - Higher expression of Aggrecan in the treated groups, except for PRP group	Bahmanpour et al., 2016 [62]
Horses Full thickness articular cartilage defect of the knee (diameter: 15 mm)	Horse APEF (Autologous Platelet-enriched Fibrin) +/− horse BMDMSCs	- Blood collection into an acid citrate dextrose bag - Isolation of fibrinogen from plasma by use of an ethanol precipitation technique - Obtainment of a fibrinogen/platelet mixture (1:1) with the thrombin solution	1-year implantation Repair tissues were evaluated by: - Arthroscopy (ICRS scores) - Histological examination - MRI - Micro-CT - Indentation tests	- No significant differences between the two groups according to arthroscopic ICRS scores - Fair-to-good fill of chondral defects and integration with the surrounding cartilage in both groups according to histological scores - Less thick cartilaginous tissue in the repair site after the addition of BMDMSCs - No variations in the stiffness of the cartilaginous tissue between the two treatments	Goodrich et al., 2016 [72]
Rabbits Chondral defect in the femoral condyle (diameter: 3 mm)	- Rabbit PRF + cartilage granules (PRFCG) *Controls:* - Rabbit PRF - Untreated cartilage defect	Preparation according to Choukroun et al., 2001 [3]	- 3-month implantation - Gross anatomy evaluation - ICRS histological scores	- Repair tissue with an intact, smooth, and hyaline-like surface resembling normal cartilage in the PRFCG group - Integration of the PRFCG implant with adjacent normal tissue, with no signs of inflammation - Histologically, better repair of the cartilage defect in the PRFCG group *versus* the PRF and untreated groups	Wong et al., 2017 [66]
Rabbits 2 mm wedge shape full-thickness defect in the medial meniscus	- Rabbit PRF fragments + defect sutured with 5–0 prolene (PRF-augmented suture group) *Controls:* - Not sutured defects (non-suture group) - Defects sutured with 5–0 prolene (suture group)	Preparation according to Choukroun et al., 2001 [3]	- 3-month implantation - Semi-quantitative histological scores	- Better morphological integrity of the meniscus in the PRF-augmented suture group than the control groups - No signs of high-grade degeneration in the PRF-augmented suture group, but mucoid changes with clear signs of degeneration in the control groups - Better healing of the meniscal defect via PRF-augmentation according to histological scores - Better congruity of articular cartilage in the PRF treated group	Wong et al., 2017 [67]
Rabbits osteochondral defect in the patellar groove (diameter: 5 mm; depth: 2 mm)	- Rabbit PRF + osteochondral autograft - Rabbit PRP + osteochondral autograft *Control:* - Osteochondral autograft	Preparation according to Choukroun et al., 2001 [3]	- 3- and 12-week implantation - ICRS macroscopic scoring system for repair evaluation - Histological examination - Immunohistochemical analysis of type-I and type-II collagen	- Macroscopical healing of the defect in the PRF group *versus* PRP and control groups at 3 weeks - Macroscopical healing of the defect with normal or nearly normal cartilage in all the 3 groups at 12 weeks - In the nongrafted portion of the defect, formation of hyaline-like cartilage in the PRF group and fibrocartilage in the other 2 groups	Maruyama et al., 2017 [73]
Rabbits Full thickness osteochondral defect in the knee joint (diameter: 5 mm; depth: 5 mm)	- Rabbit i-PRF - Rabbit PRP *Control:* - Untreated defect	- Blood collection without anticoagulant - Centrifugation (60 X g, 3 min) with Choukroun PRF Duo Centrifuge (Process for PRF, Nice, France) - Collection of the upper plasma layer designated as i-PRF	- 4- and 12-week treatment - ICRS macroscopic scoring system - ICRS histological scoring - Safranin O/fast green staining of to assess GAG content	- At 4 weeks, higher macroscopic IRCS scores in the i-PRF group in comparison with PRP and control groups, with formation of white opaque tissue well integrated with the surrounding healthy cartilage - At 12 weeks, no significant macroscopic differences among all groups - Higher ICRS histological scores in the i-PRF group, revealing complete regeneration of the cartilage and subchondral bone, with complete integration to normal tissues and identification of normal chondrocytes	Abd El Raouf et al., 2017 [61]
Dogs Osteochondral defect in the femoral condyle (diameter: 6 mm; depth: 5 mm)	- Dog PRF seeded with dog BM-MSCs *Control:* - Untreated defect	Preparation according to Choukroun et al., 2001 [3]	- 4-, 16- and 24-week implantation - ICRS evaluation score for macroscopic analysis - O’Driscoll histological grading scale for microscopic studies	- Consistently better integration of the repair tissue in the treated group *versus* the untreated control according to macroscopic scoring results - Formation of fibrous tissue in both experimental groups at 4 weeks - Histological detection of chondrocyte-like cells and cartilaginous ECM in the treated group at 16 and 24 weeks - Significantly higher histological scores in the treated group	Kazemi et al., 2017 [74]
Pigs Osteochondral defect in the femoral condyle (diameter: 8 mm; depth: 5 mm)	- Pig PRF +/- autologous cartilage fragments - Autologous cartilage fragments *Control:* - Untreated defect	- Blood collection with clot activator and gel - Centrifugation (1,066 X g, 10 min) - Separation of the jelly-like PRF from the gel-clot without the red blood cells sinking to the bottom of the tube	- 6-month implantation - Gross appearance of coverage, tissue color, defect margins, and surface - ICRS histological grading score	- Significantly better healing and repair tissue integration in the PRF+cartilage group in comparison with other 3 groups - Significantly greater histological scores in the PRF+cartilage group, with smooth repaired hyaline-like cartilage containing columnar arrangements of chondrocytes and integration of the regenerated tissue with the normal hyaline cartilage as well as the underlying subchondral bone	Sheu et al., 2017 [75]
Rabbits Osteochondral defect in the femoral condyle (diameter: 3 mm; length: 2 mm)	- Rabbit PRF releasates (PRFr) +/− autologous bone marrow-derived MSCs - Autologous bone marrow-derived MSCs *Control:* - Untreated defect	- Blood collection into a serum separation tube - Centrifugation (3,000 rpm, 10 min) - Obtainment of a fibrin clot (PRF) between a clear yellow serum layer and a coagulated red blood cell layer	- 12-week treatment - Gross assessment of shape, color, contour, and uniformity of the cartilage - Histological scoring system	- Decrease of the defect size and increase of the regenerated cartilage volume in the PRFr+MSCs group - Better histological indices (i.e., matrix deposition, cell distribution, and tissue surface) in the PRFr+MSCs group - Thicker hyaline-like cartilaginous tissue with normal GAG production in the PRFr+MSCs group in comparison with other 3 groups	Wu et al., 2017 [53]
Rabbits Osteochondral defect in the femoral condyle (diameter: 3 mm; length: 2 mm)	- Rabbit PRF releasates (PRFr) +/− autologous ADSCs - Rabbit PRFr + chondrocytes - Autologous ADSCs *Control:* - Untreated defect	Preparation according to Wu et al., 2017 [53]	- 14-week treatment - Gross investigation of defect filling, integration to border zone and macroscopic appearance of the implant - ICRS histological grading score	- Decrease of the defect size and increase of the repaired cartilage volume in the PRFr+ADMSCs group - Better matrix, cell distribution, and surface indices in the PRFr+ADSCs group than other groups according to histological grading scores - Thicker hyaline cartilage-specific ECM in the PRFr+ADMSCs group - Similar histological scores for ADSCs and PRFr groups	Hsu et al., 2018 [76]
Rabbits Full thickness cartilage defect of the ear (5 × 5 × 1 mm)	- Rabbit PRF +/- allogenic ADSCs - Allogenic ADSCs *Control:* - Untreated defect	Preparation according to Choukroun et al., 2001 [3]	- 1-, 2-and 3-month implantation - Macroscopic evaluation - Histological analysis - Gene/protein expression study of type-II collagen - Immune response evaluation by determining blood levels of CD4/CD8, IL-2 and IL-4	- Best rate of repair at all observation points in the PRF+ADSCs group, with 90% greater repair rate than other groups at 3 months - More efficient repair of the cartilage defect in the PRF+ADSCs group, with the treated area almost completely filled by naïve chondrocytes. - Higher type-II collagen expression, both at the gene and protein levels, in the PRF and PRF+ADSCs groups - No significant immune response induced by allogenic ADSC transplantation	Xu et al., 2018 [77]

ADSCs, Adipose-derived Stem Cells; APEF, Autologous Platelet-enriched Fibrin; BMDMSCs, Bone Marrow-derived Mesenchymal Stem Cells; BM-MSCs, Bone Marrow-derived Mesenchymal Stem Cells; ECM, Extracellular Matrix; GAG, glycosaminoglycan; ICRS, International Cartilage Repair Society; IL, Interleukin; i-PRF, injectable Platelet-rich Fibrin; L-PRF, Leukocyte- and Platelet-rich Fibrin; L-PRP, Leukocyte- and Platelet-rich Plasma; micro-CT, micro-Computed Tomography; MRI, Magnetic Resonance Imaging; PRF, Platelet-rich Fibrin; PRFM, Platelet-rich Fibrin Matrix PRFr, Platelet-rich Fibrin releasates; PRP, Platelet-rich Plasma; rhSDF_1_, recombinant human Stromal cell-derived Factor 1; +/−, with or without.

**Table 3:** Clinical application of PRF for cartilage repair.

End Use Destination	Hemocomponent/Experimental Groups	PRFPreparation Protocol	Characterization Parameters	Major Findings	Reference
Hemophilic ankle arthropathy (focal lesions)	*n* = 5 patients (mean age = 33 ± 6.78 years): collagen membrane loaded with BMDCs and PRF	Preparation according to the Vivostat^®^ system	Mean follow up: 2 years The postoperative outcome was evaluated by: - AOFAS scores - radiographs - MRI and Mocart scores	- All patients showed complete filling of the talar defect - The implant borders were completely/partially integrated with the adjacent cartilage - In all patients presented inhomogeneous, hyperintense repair tissue was detected - Three patients had subchondral bone edema or cyst - Overall, the data showed good osteochondral regeneration and no progression of joint degeneration	Buda et al., 2015 [82]
Knee cartilage focal lesions	*n* = 15 patients: microfractures and PRF; *n* = 16 patients: microfractures and PRP; *n* = 17 patients: microfractures alone	-	Follow up: 2, 5 years Postoperative evaluation of patients was performed by: - clinical scores (i.e., IKDC, VAS pain) - MRI and Mocart scores	- Platelet concentrates allowed to achieved better clinical results compared to microfracture alone - The PRF was more effective than the PRP at 2 years, with loss of significance at 5 years - According to Mocart score, PRF gave better results earlier than the other two treatments	Papalia et al., 2016 [84]
Knee cartilage focal lesions	*n* = 25 patients (mean age = 29 ± 7.3 years): single-step AMIC procedure based on microfracture and application of autologous PRF called CLP-MB membrane, combined with an injectable collagen scaffold (Cartifill)	- Blood collection by apheresis - Separation of CLP and plasma - Cryoprecipitate formation from freeze/thawed plasma - Mixing of CLP and cryoprecipitate (CLP mix) - Activation of the CLP mix with calcium gluconate - Incubation at 37 °C for 10 min - Centrifugation (7333× *g*, 25 min)	Pre-implant characterization: - assessment of blood cell composition, CD34^+^/CD133^+^/VEGFR2^+^ cell content, fibrinogen concentration during each preparation phase - release of PDGF-AB, TGF-β1 and VEGF -mechanical tests Clinical trial: Follow-up: 1, 6 and 12 months Patients were evaluated by: - NMR and/or radiographic scans - VAS pain - IKDC scores	- Quality control tests during each phase of CLP-MB preparation assured for the obtainment of a standardized, traceable and safe product - The treatment with the hemocomponent provided short-term pain relief and functional improvement	D’Antimo et al., 2017 [85]
Rhinoplasty (dorsal nasal augmentation)	*n* = 19 patients: cartilage scales-cartilage pâté compound graft with PRGF *n* = 21 patients: cartilage scales-cartilage pâté compound graft with i-PRF *n* = 8 patients: cartilage pâté graft with a-PRF	Preparation according to Choukroun et al., 2001 [3]	Follow-up controls every 3 months Medical records to assess the surgical outcome included: - follow-up notes - pre- and post-operative photographic documentation	- Satisfactory dorsal nasal augmentation in 47 patients - 1 mm-horizontal displacement of the graft in one patient 3 months after surgery, with no tendency for further displacement - No dorsal irregularities, nor signs of resorption, erythema, inflammation	Kovacevic et al., 2017 [86]

a-PRF, advanced PRF; AOFAS scores, American Orthopedic Foot and Ankle Society scores; BMDCs, Bone Marrow-derived Cells; CLP, leukocyte and platelet concentrate; CLP-MB, leukocyte- and platelet-rich fibrin membrane; i-PRF, injectable PRF; IKDC, International Knee Documentation Committee; MRI, Magnetic Resonance Imaging; NMR, Nuclear Magnetic Resonance; PRF, Platelet-rich Fibrin; PRGF, Platelet-rich Growth Factors; PRP, Platelet-rich Plasma; VAS, Visual Analog Scale.

**Table 4:** In vitro studies on PRF and tenogenesis.

Hemocomponent/Experimental Groups	PRF Preparation Protocol	Characterization Parameters	Major Findings	Reference
- Human PR-matrix - Human PP-matrix - Human purified fibrin	- Blood collection into 3.8% (wt/vol) sodium citrate - Centrifugation at 4 °C: (a) PR-plasma → 460× *g*, 8 min (b) PP-plasma → 4500× *g*, 12 min - Platelet counts before clotting - Addition of calcium chloride at a final concentration of 22.8 mM	- Proliferation of human tenocytes - Secretion of TGF-β1, VEGF and HGF (+/− cells) - Synthesis of type-I collagen (Coll-I)	- Significantly increased platelets cells proliferation - Increase in Coll-I synthesis with any difference between PR- and PP-matrices - Higher levels of TGF-β1 in PR-matrix samples (i.e., +/− tenocytes) than PP-matrices - Increased synthesis of VEGF and HGF by tenocytes on fibrin matrices - Significantly higher levels of VEGF, but not HGF, in presence of platelets	Anitua et al., 2006 [101]
- Dog PRF matrix - Dog PRF membrane - Dog whole blood clot	a) PRF matrix - Blood collection in tube with trisodium citrate - 1st centrifugation (1100× *g*, 6 min) - Supernatant transfer in a tube with calcium chloride - 2nd centrifugation (1450× *g*, 15 min) b) PRF membran e- Blood collection in tube with trisodium citrate and the proprietary separator gel. - 1st centrifugation (1100× *g*, 6 min) - Supernatant transfer in a glass vial with calcium chloride - 2nd centrifugation (4500× *g*, 25 min) - Suspension of the resulting membrane in serum	- Quantification of eluted TGF-β1 - Evaluation of the mitogenic effect on canine tenocytes	- Both PRF constructs release significantly higher levels of TGF-β1 than blood clot, significantly increasing cell proliferation - Significantly higher levels of TGF-β1 were released from PRF membrane than PRF matrix, significantly increasing cell proliferation	Visser et al., 2010 [102]
- Human standard/gelatinous L-PRF - Human dry/compressed L-PRF	- Blood collection (at 8.30 am.) incitrate tubes - Centrifugation for 12 min with different G-forces: (1) 200× *g*, (2) 400× *g*, (3) 1000× *g* - Count of platelets, leukocytes and red blood cells in extracted supernatant and “buffy coat” *versus* normal blood	- Leukocyte content - Release of GFs (i.e., TGF-β1, VEGF, MPO, IGF1, PDGF-AB, CXCL4) - Relationship between matrix preparation methods and GFs concentrations	- Highest concentration of platelets and leukocytes with 400× *g* centrifugation - L-PRF clots showed in vitro a continuous release of GFs which were significantly higher than levels expressed by normal blood at each culture time point - Higher release of GFs (i.e., CXCL4, IGF-1, PDGF-AB, and VEGF) by the standard/gelatinous- compared to the dry/compressed group	Zumstein et al., 2012 [111]
- Human PRF-matrix - Fibrin matrix based on PRP (ViscoGel; Arthrex, Naples, FL) *Controls:* - Human highly cross-linked collagen membrane (Arthroflex; LifeNet Health, Virginia Beach, VA) - Porcine non-cross-linked collagen membrane (Mucograft; Geistlich Pharma, Lucerne, Switzerland) - Human fresh-frozen rotator cuff tendon (allograft)	- Blood collection - Centrifugation (3000 rpm, 10 min)	- Differentiation, proliferation of human MSCs	- MSCs successfully differentiated into all cell lines - A significantly greater number of cells adhered to both the non-cross-linked porcine collagen scaffold and PRF-matrix - Significantly higher proliferation in the non-cross-linked porcine collagen scaffold vs PRF-matrix and fibrin matrix based on platelet-rich plasma - No significant differences at the live/dead assay	Beitzel et al., 2014 [103]

Coll-I, type-I collagen; CXCL4, Platelet Activity Factor; GFs, Growth Factors; HGF, Hepatocyte Growth Factor; IGF-1, Insulin Growth Factor-1; L-PRF, Leukocyte- and Platelet-Rich Fibrin; MPO, Myeloperoxidase; MSCs, Mesenchymal Stem Cells; PDGF-AB, Platelet-Derived Growth Factor-AB; PP, platelet-poor; PR, Platelet-rich; PRF, Platelet Rich Fibrin; TGF-β1, Transforming Growth Factor-beta1; VEGF, Vascular Endothelial Growth Factor; vs, versus. +/−, with or without.

**Table 5:** Pre-clinical studies on PRF for tendons repair.

End Use Destination	Hemocomponent/Experimental Groups	PRF Preparation Protocol	Characterization Parameters	Major Findings	Reference
Sheeps Achilles tendon injected at 2.5 cm proximal to the bone insertion	- Injection of autologous sheep calcified PR-plasma - Injection of autologous sheep PP-plasma - Injection of saline	- Blood collection into 3.8% (wt/vol) sodium citrate - Centrifugation at 4 °C: (a) PR-plasma → 460× *g*, 8 min (b) PP-plasma → 4500× *g*, 12 min - Platelet counts before clotting - Addition of calcium chloride at a final concentration of 22.8 mM	- Cell density, morphology and distribution - Vascularization - Inflammation	- Higher increase in cell density in the fascicles treated with PR- and PP-plasma - Ovoid but aligned cells in PR- and PP- treated tendons - Neovascularization is promoted with both PR-and PP-plasma - No inflammatory cells in both PR-and PP-plasma treatment	Anitua et al., 2006 [101]
Sheeps acute model of Achilles tendon rupture	- Re-approximation of the tendon ends with suture only - Re-approximation with suture augmented with ADP wrapped around the repair and sutured to the tendon - ADP wrapped around the proximal and distal margins of the tendon, bridging a 1.5 cm gap, with autologous PRPFM sutured in place within the gap	- PRPFM—Cascade Autologous Platelet System-4, Musculoskeletal Transplant Foundation	- Mechanical tests - Cell and tissue morphology - Vascularization - Scaffold incorporation- Inflammation	- Significant difference in elongation between the operated limb vs unoperated limb in suture only group and ADP + PRPFM group but not in suture + ADP group - No apparent fibrosis in all groups - Increased tendon thickness in suture only group - New tendon fibers without increasing tendon thickness (2/6 animals) in suture + ADP group - Complete bridging of the gap, with no change in tendon thickness in ADP + PRPFM (2/6 animals) - Peripheral integration of the APD to tendon fibers - APD +/− PRPFM augments Achilles tendon repair	Sarrafian et al., 2010 [114]
Dogs patellar tendon; sharp incision of the central third	- Autologous dog PRF membrane to fill the injury site - Surgical closure following resection of the central third of the patellar tendon	- Blood collection in tubes with trisodium citrate and a separator gel. - 1st centrifugation (1100× *g*, 6 min) - Transfer of PRP supernatant in a vial containing 1.0 M calcium chloride. - 2nd centrifugation (4500× *g*, 25 min) while fibrin polymerization ensued	- Gross healing assessment and cross-sectional area - Cell density - Vascularization - Collagen and GAG	- Repair tissue in both groups - No histological significant difference (i.e., cellularity, vascularity, collagen organization, or GAG content) - Hypercellular fibrovascular repair tissue in defect site of both groups - Significantly greater cross-sectional area of PRF membrane–treated tendons vs the control group - PRF membrane did not enhance the rate/quality of tendon healing but it increases repair tissue surrounding the defect.	Visser et al., 2011 [117]
Rabbits Toe flexor tendon; sharp transection between the A1 and A2 pulley and immediate surgical repair	- Allogenic PRP - Allogenic PRP-F matrix - Commercial fibrin (Beriplast P Combi Set; CSL Behing K.K., Tokyo, Japan) *Control:* Natural healing of the repair site	- Blood collection in syringe with acid citrate dextrose-A - 1st centrifugation (2400 rpm, 10 min at 4 °C) - 2nd centrifugation of plasma (3600 rpm, 10 min at 4 °C) - Platelets count - Addition of fibrin matrix (Beriplast P Combi-Set; CSL Behring K.K., Tokyo, Japan): liquid A (0.25 µL) + liquid B (0.25 µL)	- Edema of the toes - Adhesions extent - Mechanical tests - Histological analysis	- No significant difference in edema/adhesion scores - Significantly increased healing strength by PRP-F matrix	Sato et al., 2012 [115]
Rabbits Experiment 1 Bone-patellar tendon-bone. Removal of the central half of each patellar tendon Experiment 2 Removal of medial collateral ligament	Experiment 1 - Allogenic rabbit CPFS; *Control:* untreated defect of the controlateral patella Experiment 2 Allogenic CPFS sheet *Control:* Insertion of rivets without reconstruction of the controlatelar medial collateral ligament	- Blood collection in tubes with a sodium citrate solution (5% wt/vol) - 1st centrifugation (3000 rpm, 15 min at 4 °C) - 2nd centrifugation of platelet poor plasma (3000 rpm, 15 min at 4 °C) - Freezing of buffy coat layer and platelet poor plasma (−80 °C) - Defrosting and enriching by ultrafiltration twice of platelet poor plasma; defrosting of buffy coat. - Blending of the two fractions and addition of calcium gluconate (final concentration 23 mM) - Incubation at 37 °C for 3 h - Pressure treatment in aqueous solution of 10 mM calcium chloride at 4 °C	- Repair tissue thickness - Mechanical tests - Inflammation	Experiment 1 - the ultimate failure load and stiffness were higher for the CPFS-treated group than untreated knee - Presence of dense and longitudinally aligned collagen bundles - No signs of immunological rejection of allogenic scaffold Experiment 2 - CPFS promoted ligament repair tissue vs the untreated side - The ultimate failure load of the CPFS repair tissue at 20 weeks was 78% of that in healthy controls of the same age CPFS enhanced/accelerated healing of tendons and ligaments	Matsunaga et al., 2013 [118]
Rats Tendon-bone insertion site, rotator cuff. Transection and transosseous suture repair of the supraspinatus tendon	- Surgical repair + allogenic PRFM *Control:* - Controlateral shoulder, only surgical repair	- Blood collection in syringe with 0.5 cc of acid citrate dextrose anticoagulant solution and thixotropic polyester separator gel. - 1st centrifugation (1500 rpm, 15 min) - 2nd centrifugation of the platelet-rich layer (3000 rpm, 6 min)	- Mechanical tests - Histological analysis (i.e., collagen tissue organization/maturation; cartilage formation	- Higher ultimate load to failure, stress, and stiffness values for experimental group repairs - No differences in biomechanical testing between the groups - Less collagen organization and cartilage formation at the insertion site in the experimental group - PRF-membrane does not recapitulate the native enthesis with exuberant/disordered healing response with fibrovascular scar tissue	Hasan et al., 2016 [108]
Rabbits Flexor digitorum profundus tendon	Part I - Autologous rabbit PRF, wrapped around the repair site, tagged with suture Part II - Autologous rabbit PRF interposed between the tendon repair ends by a 2-strand repair *Control:* Control tendons	- Blood collection without anticoagulant - Centrifugation (2700 rpm, 12 min at room temperature) - Compression of the PRF clot	- Range of motions analysis - Cross-sectional area - Mechanical tests	- No significant increase in range of motion - Significant increase in cross-sectional area of the tendons in the PRF group - The control had a higher load and stress to failure but similar stiffness and modulus to the PRF groups - The PRF did not have a major influence on cellular organization - Undesirable effect on the biomechanical properties of repaired flexor tendons	Liao et al., 2017 [116]

ADP, Acellular Porcine Dermal patch; CPFS, Compact Platelet-rich Fibrin Scaffold; GAGs, Glycosaminoglycan; PLTs, Platelets concentration; PP, Platelet-Poor; PR, Platelet-rich; PRFM, Platelet Rich Fibrin Matrix; PRP-F matrix, Platelet-Rich Plasma and Fibrin matrix; vs, versus; WB, Whole Blood; +/−, with or without.

**Table 6:** Clinical trials on PRF derivatives for tendons repair.

End Use Destination	Hemocomponent/Experimental Groups	PRF Preparation Protocol	Characterization Parameters	Major Findings	Reference
Full-thickness rotator cuff tear	*n* = 20 patients (mean age = 57.6 years): Arthroscopic single-row rotator cuff repair + 2 autologous PRP, sutured into the repair site *n* = 20 patients (mean age = 57.8 years; range = 44–69 years): Arthroscopic single-row rotator cuff repair	- Cascade autologous platelet system	Mean follow up and range: PRP, 28.3 (24–44) months; no PRP, 33 (24–44) months - MRI - Clinical outcome measures by ASES, Rowe, SANE, SST and Constant scores	- Retears: with PRP: 6 of 20 (30%); no PRP: 12 of 20 (60%) - Cuff tear size (no. healed): <3 cm, 7 of 14 (50%) no PRP; 12 of 14 (86%) with PRP. ≥3 cm, 1 of 6 (17%) no PRP; 2 of 6 (33%) with PRP - Significant clinical differences showing lower re-tear rates by MRI only with Rowe score	Barber et al., 2011 [104]
Arthroscopic rotator cuff repair	*n* = 43 patients (mean age = 55.5 years): Tear size (cm): small (<1); medium (1–3), PRFM *n* = 45 patients (mean age = 55.2 years): Tear size (cm): small (<1), medium (1–3), no PRFM	- Blood collection in a tube with trisodium citrate and a thixotropic polyester separator gel - 1st centrifugation (1100 rpm, 6 min) - Transfer of the supernatant in a bottle containing calcium chloride (1.0 M) - 2nd centrifugation (4500 RCF, 25 min)	Follow up: 16 months - Clinical outcome by Constant scores - MRI	- Statistically significant improvement in both groups but any among groups - Difference in alterations of MRI signal intensity - Re-rupture in 10.5% patients of control group and 2.5% in PRFM group but any additional treatment occurred - No difference in tendon thickness or in size of the tendon footprint tendon thickness	Castricini et al., 2011 [107]
Arthroscopic rotator cuff repair	*n* = 16 patients (mean age = 65 ± 7 years): Tear size: 3.8 ± 1.1 cm, PRFM *n* = 21 patients (mean age = 65 ± 9 years): Tear size: 3.9 ± 1.1 cm, no PRFM	- Blood collection in a tube with trisodium citrate - 1st centrifugation (1100 rpm, 6 min) - Transfer of supernatant into a second tube containing calcium chloride, which initiates the fibrin-clotting cascade - 2nd centrifugation (1450 rpm, 15 min) The Cascade Autologous Platelet System was used to prepare the PRFM	Mean follow-up: PRFM group, 13 ± 4 months; Untreated group, 27 ± 8 months - Operative time - MRI - Clinical outcome scores by Constant, WORC, SANE, ASES, UCL	- Retear rates: statistically significantly higher in the PRFM group (56.2%) vs. controls (38.1%) - Functional outcome scores postoperatively: not significantly improved in PRFM vs. controls - Operative time (min): 152 ± 31 in PRFM group vs 161 ± 40 in control group - 2 infections in the PRFM group - The augmentation of at-risk rotator cuff tears with PRFM did not result in improved retear rates or functional outcome scores compared with controls	Bergeson et al., 2012 [106]
Full-thickness rotator cuff tear	*n* = 40 patients (mean age = 58.90 ± 9.86 years): Tear size (nr. of patients): small: 10; medium: 20; large: 10, PRFM treatment *n* = 39 patients (mean age = 7.21 ± 9.42 years): Tear size (nr. of patients): small: 10; medium: 19; large: 10, No PRFM	- Cascade Membrane (Musculoskeletal Tissue Foundation, Edison, NJ, USA)	Follow-up: 6 weeks, 3 and 12 months - Power doppler ultrasound - Manual muscle testing ratio - Clinical outcome scores by ASES and l’Insalata - Strength measurements using a handheld dynamometer	- Intact repair in 24 of 36 (67%) in the PRFM group and 25 of 31 (81%) in the control group - No differences in tendon-to-bone healing - No demonstrable effect on tendon healing vascularity, manual muscle strength, or clinical rating scales by PRFM - Negative effect of PRFM on healing according to regression analysis	Rodeo et al., 2012 [109]
Arthroscopic rotator cuff repair	*n* = 30 patients (mean age = 59.67 ± 8.16 years): Tear size: 1.77 ± 0.84 cm, PRFM treatment (commercially available) *n* = 30 patients (mean age = 64.50 ± 8.59 years): Tear size: 1.72 ± 1.18 cm, no PRFM	- Cascade Membrane (Musculoskeletal Tissue Foundation, Edison, NJ, USA)	Follow-up: 1 h, 3, 6, 9, and 12 weeks, 1 year - Operative time - VAS pain scores, ROM, SST, FF, ER, UCLA, ASES scores - Narcotic consumption - MRI	- No complications - Longer mean surgery time for the PRFM group than control group - No significant difference in VAS, ROM, SST, FF, ER or ASES scores or narcotic use - Similar UCLA scores in both groups at baseline but statistically significantly lower in the PRFM group at follow-up - No differences in MRI - No significant improvement in perioperative morbidity, structural integrity or clinical outcome in PRFM in early follow-up	Weber et al., 2013 [110]
Full-thickness rotator cuff tears	*n* = 20 patients (mean age = 55 years): Tear size: ≤3 cm in anteroposterior length, suture-bridging double-row repair + PRPFM *n* = 20 patients (mean age = 57 years): Tear size: no greater than 3 cm in anteroposterior length, triple - loaded single row repair + PRPFM	- Cascade Membrane (Musculoskeletal Tissue Foundation, Edison, NJ, USA)	Mean follow-up and range: double-row group, 27 (12–46) months; single-row group, 28 (12–49) months - ASES, Rowe, SST, Constant, SANE - MRI	- No statistical difference on clinical outcome scores between groups - No MRI difference in rotator cuff tendon re-tear rate (i.e., 15% in both groups)	Barber et al., 2016 [105]
Arthroscopic rotator cuff repair	*n* = 17 patients (mean age = 65 years): Tear size (area): 322 ± 180 mm^2^, L-PRF *n* = 18 patients (mean age = 66 years): Tear size (area): 445 ± 421 mm^2^, No L-PRF	- Blood collection (at 8.30 am.) incitrate tubes - Centrifugation for 12 min with different G-forces: (1) 200× *g*, (2) 400× *g* and (3) 1000× *g*. - Count of platelets, leukocytes and red blood cells in extracted supernatant and “buffy coat” vs normal blood	- Mean follow-up: L-PRF, 14 months; Untreated group, 15 months - SSV, VAS for pain, SST, Constant-Murley	- No complications in either group - No significant differences in clinical outcome, healing rate, mean postoperative defect size, and tendon quality at 12 mo follow-up	Zumstein et al., 2016 [112]
Acute rupture of Achilles tendon	*n* = 11 patients (mean age = 32.5 ± 3.4 years): PRF augmentation *n* = 9 patients (mean age = 34.5 ± 3 years): No PRF *n* = 8 patients (mean age = 30 ± 4.4 years): Healthy	- Blood collection in a tube with sodium citrate - Centrifugation (3000 rpm, 10 min) The protocol included specific jellifying agents (i.e., calcium gluconate and batroxobin)	Follow-up: 6 months - Gait analysis	- % of the stance time of the operated leg, double-support time of the healthy leg, network of the ankle during the gait cycle showed statistically significant differences between the no-PRF and the healthy group - No differences between PRF and healthy groups - Suture + PRF augmentation shows significant functional improvements in motion efficiency	Alviti et al., 2017 [113]
Gluteus medius tendons	*n* = 18 patients (mean age = 60.26 ± 8.8 years): Tear size: small or low-grade partial tear (33.3%); large or high-grade partial tear (50.0%); large or high-grade full tear (16.7%), PRFM *n* = 29 patients (mean age = 63.09 ± 12.0 years): Tear size: small or low-grade partial tear (31.0%); large or high-grade partial tear (58.6%); large or high-grade full tear (10.4%), no PRFM	-	Follow-up: 1 year - Demographic variables	- No effect of PRFM on repair in terms of pain or clinical evidence of retears - PRFM may have a role in improving subjective outcomes of overall and hip-specific physical functioning	Saltzman et al., 2018 [119]	

ASES, American Shoulder and Elbow Surgeons, Rowe; ER, external rotation; FF, Forward Flexion; L-PRF, leucocyte- and platelet-rich fibrin; PRFM, Platelet Rich Fibrin Matrix; PRPFM, Platelet Rich Plasma Fibrin Matrix; ROM, Range Of Motion; SANE, Single Assessment Numeric Evaluation; SST, Simple Shoulder Test; SSV, Subjective Shoulder Value; UCLA, University of California, Los Angeles; VAS, Visual Analog Scale; WORC, Western Ontario Rotator Cuff Index.
